# Causal pathways of plasma metabolites: unveiling metabolic associations in liver diseases

**DOI:** 10.1186/s12986-025-01017-9

**Published:** 2025-11-03

**Authors:** Xiaoxuan He, Yanfen Ma, Qian Wu, Jing Feng, Wei Wang, Shan Zhao, Xiaoqin Wang

**Affiliations:** Department of Clinical Laboratory, The First Affiliated Hospital of Xi’an Jiaotong University, Xi’an, 710061 Shaanxi Province China

**Keywords:** Plasma metabolites, Liver fibrosis, Liver cirrhosis, Hepatoma, Two-Sample mendelian randomization, Causal analysis

## Abstract

**Background:**

This study aimed to explore the causal relationships between 1,400 plasma metabolites and various chronic liver conditions, including liver fibrosis or cirrhosis, liver carcinoma, hepatic failure, and metabolic liver disorders.

**Methods:**

This study used a two-sample Mendelian randomization (MR) method. Forward analysis focused on associations with four liver disease categories, whereas reverse analysis examined links with specific metabolites or ratios. Five MR methods were applied, primarily the inverse variance weighted (IVW) method. The IVW, Egger, and Weighted Median analyses showed p-values < 0.05 with positive directions, suggesting strong positive effects. The IVW analysis alone indicated a potential positive effect with p-value < 0.05. Sensitivity analyses ensured the results’ robustness and validity.

**Results:**

In the forward causal analysis, we identified 252 potential causal associations, 13 of which were notably strong. Specifically, six plasma metabolites and metabolic ratios were found to increase the risk of fibrosis/cirrhosis, while the other three were associated with an increased likelihood of liver cancer. Additionally, three were positively correlated with metabolic liver disease risk and one was inversely related to liver failure risk. In contrast, reverse causal analysis revealed that fibrosis/cirrhosis was linked to ten plasma metabolites and metabolic ratios, whereas liver cancer was associated with three plasma metabolites and metabolic ratios.

**Conclusion:**

Our findings underscore the significant association between an elevated glucose-to-mannose ratio and a range of liver conditions, including fibrosis/cirrhosis, metabolic liver disease, and hepatoma, thereby highlighting the crucial role of glucose metabolism in liver disease.

**Supplementary Information:**

The online version contains supplementary material available at 10.1186/s12986-025-01017-9.

## Introduction

Chronic liver disease, characterized by hepatocyte damage and impaired liver function, poses a significant clinical burden [1]. The major etiological factors include viral hepatitis, excessive alcohol consumption, drug-induced liver injury, metabolic disorders, and autoimmune hepatitis. As the disease advances, patients often experience systemic manifestations that drastically diminish their functionality and quality of life [2]. Without prompt treatment, chronic liver inflammation can evolve into liver fibrosis, which may subsequently progress to cirrhosis. This progression exacerbates liver dysfunction and considerably increases the risk of hepatic failure and hepatocellular carcinoma (HCC).

Liver fibrosis is a common liver disorder caused by the activation of hepatic stellate cells (HSC) due to factors such as viral infections and alcohol abuse [3, 4]. This activation leads to excessive deposition of the extracellular matrix (ECM) [5, 6]. If untreated, fibrosis can progress to liver cirrhosis, the 11th leading cause of death globally, causing about 2 million deaths annually [7, 8]. Mechanisms driving this progression include HSC activation, transforming growth factor-beta (TGF-β) signaling, and a persistent wound-healing response. Hepatoma is a significant cause of cancer-related deaths worldwide, ranking fourth and increasing steadily each year [9]. HCC accounts for approximately 75% of all hepatoma cases and is dangerous because it often lacks early symptoms, progresses rapidly, and has a high risk of spreading [10]. Owing to limited effective treatments, HCC usually leads to poor outcomes and low survival rates [11–13]. Additionally, metabolic liver disease, which includes non-alcoholic fatty liver disease (NAFLD), inherited metabolic disorders, and liver damage from metabolic syndrome, is becoming a major global health issue because of its links to insulin resistance, obesity, diabetes, and dyslipidemia, and its progression involves lipid accumulation, inflammation, oxidative damage, and fibrosis [14].

Plasma metabolites are direct indicators of hepatic metabolic activity and reflect functions such as energy metabolism, detoxification, and bile acid regulation. They are also crucial markers for liver health. Chronic liver diseases can develop without noticeable symptoms early on but can lead to severe complications, such as hepatic failure, if not treated promptly. Early diagnosis and treatment are key to improving patient outcome. Studies have found links between specific plasma metabolites and conditions such as autoimmune hepatitis, cholestatic liver disease, and HCC [15–17]. However, the exact causes and effects of plasma metabolites in chronic liver diseases, including fibrosis, cirrhosis, hepatoma, hepatic failure, and metabolic liver dysfunction, are not fully understood.

Mendelian randomization (MR) analysis is a reliable epidemiological method used to identify causal links between risk factors and diseases [18]. It uses genetic variants that are randomly assigned at birth as tools to reduce biases from other factors and reverse causation. In this study, large-scale genome-wide association study (GWAS) data were used to perform two-sample MR analysis. This analysis aimed to discover potential causal relationships between large-scale metabolomics and various chronic liver diseases such as liver fibrosis and cirrhosis, hepatoma, hepatic failure, and metabolic liver dysfunction. Our findings were confirmed using multiple MR methods and sensitivity analyses, highlighting their potential as biomarkers and therapeutic targets for further research and development.

## Materials and methods

### Study design and SNP selection

**Fig. 1:**
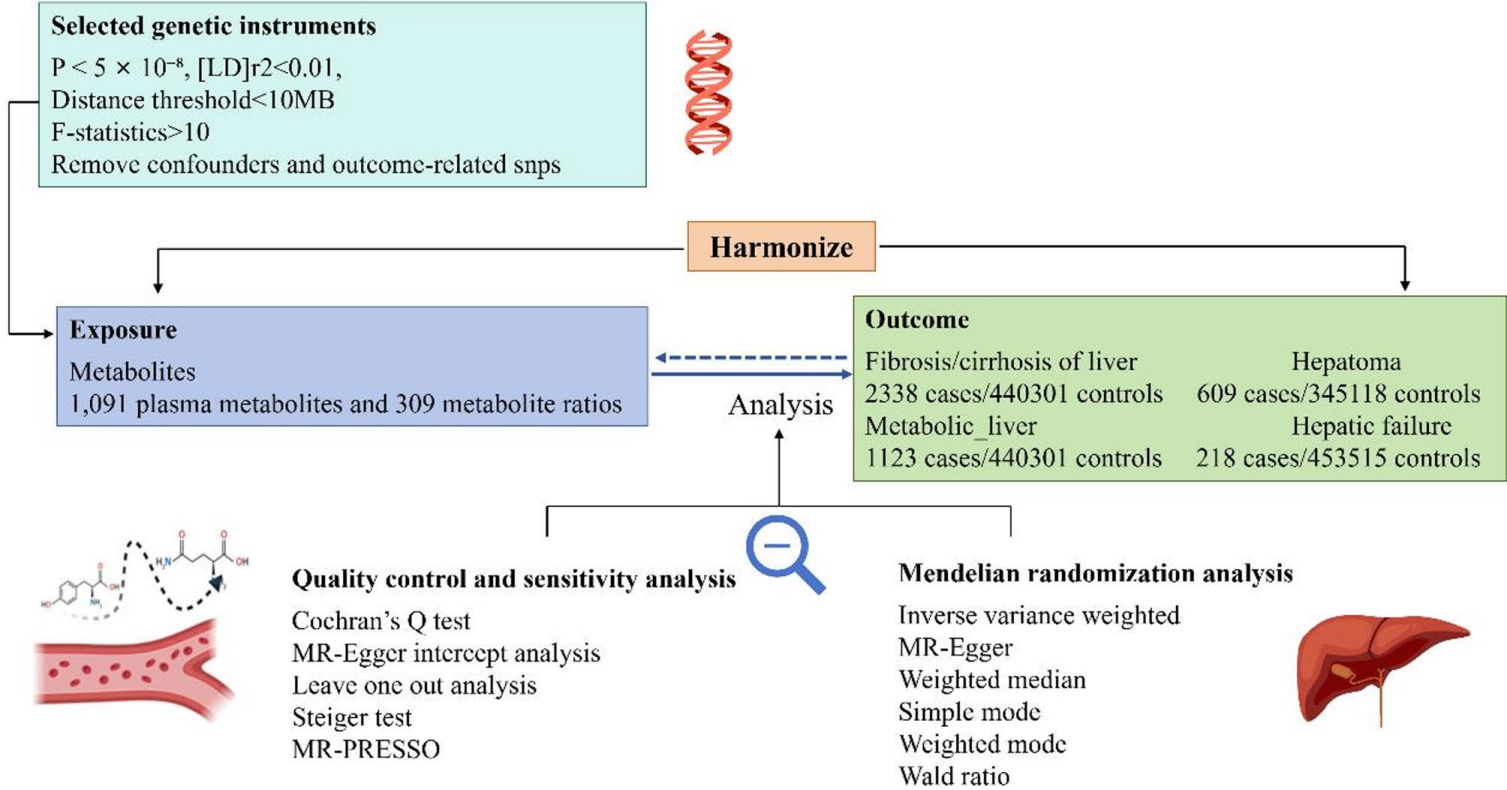
The flowchart of the process in this study

This study employed a two-sample MR approach, as depicted in Fig. 1, to investigate the causal associations between plasma metabolites and five subtypes of liver disease: liver fibrosis and cirrhosis, hepatoma, hepatic failure, and metabolic liver disease. The research design encompassed two primary phases: forward and reverse MR analysis. During forward MR analysis, 1,091 plasma metabolites and 309 metabolic ratios were scrutinized as exposure variables, with the five liver disease subtypes acting as the outcomes. Conversely, reverse MR analysis evaluated the liver disease subtypes as exposures to ascertain their causal impacts on plasma metabolites and metabolic ratios.

To ensure the validity of the genetic instrumental variables (SNPs, single nucleotide polymorphisms), we enforced strict criteria: SNPs had to show genome-wide significant associations with exposure variables (*P* < 5 × 10⁻⁸). Independence among SNPs was confirmed through linkage disequilibrium (LD) analysis, with only SNPs having r^2^ < 0.01 within a 10 MB window retained to prevent collinearity. The strength of each SNP was assessed using F-statistics, with values above 10 considered robust (calculated as R² × (N − k − 1)/[(1 − R²) × k], where R² = [2 × β² × (1 − EAF) × EAF]/[2 × β² × (1 − EAF) × EAF + 2 × N × SE² × (1 − EAF) × EAF], N is the GWAS sample size, k is the number of SNVs, β is the genetic effect, EAF is the effect allele frequency, and SE is the standard error of β). Additionally, the Steiger test was used to minimize reverse causality and strengthen instrumental variable selection. To maintain consistency, the selected SNPs were harmonized and those associated with outcome variables or identified as confounders were excluded, enhancing the reliability and validity of causal inferences.

The selected SNPs were harmonized to maintain consistency between the exposure and outcome datasets. SNPs that were identified as potential confounders or associated with outcome variables were excluded from subsequent analysis, thereby enhancing the reliability and validity of the causal inferences drawn.

### Data sources

The GWAS data for human plasma metabolites utilized in this study originated from the analysis conducted by Chen Y et al. in the Canadian Longitudinal Study on Aging (CLSA), which included 8,299 European participants (PMID: 36635386) [19]. This study investigated the genetic associations of 1,091 metabolites and 309 metabolic ratios, as detailed in Table S1. The comprehensive dataset is publicly accessible through the GWAS Catalog (GCST90199621-GCST90204063) at https://www.ebi.ac.uk/gwas/.

Summary statistics for the four liver disease outcomes were sourced from the Finn-Gen consortium, which integrates genetic data with Finnish population registries. Specifically, the Finn-Gen R9 release (https://r9.finngen.fi/) was used, comprising the following datasets: liver fibrosis and cirrhosis with 2,338 cases and 440,301 controls, hepatoma with 609 cases and 345,118 controls, hepatic failure with 1,123 cases and 444,301 controls, and metabolic liver disease with 218 cases and 453,515 controls.

### Statistical analysis

This study utilized R 4.3.1, and the two-sample MR package (v. 0.5.5) to perform analyses, ensuring proper alignment of allelic orientation between exposure and outcome instrumental variables. Missing variants were substituted with proxy SNPs (r^2^ ≥ 0.8), whereas unmatched SNPs were excluded from the analysis. MR analyses were conducted using multiple methods, including Inverse Variance Weighted (IVW), MR-Egger, Simple Mode, Weighted Median, Weighted Mode, and Wald Ratio. IVW was employed as the primary analytical method, whereas the other approaches provided complementary evaluations of the causal effects. The Wald ratio method is applicable when a single IV is available. This method determines causal effects by directly calculating the ratio of the estimated effect size of the exposure variable to that of the outcome variable. The results of the IVW, Egger, and Weighted Median analyses consistently demonstrated P-values below 0.05, with uniformly positive effect directions, suggesting a strong positive association. Specifically, an IVW P-value below 0.05 indicates the likelihood of a positive effect. The ratio method is applicable when a single instrumental variable is available. This method determines causal effects by directly calculating the ratio of the estimated effect size of the exposure variable to that of the outcome variable.

Sensitivity analyses were performed using Cochran’s Q test to ensure the validity and robustness of the findings. Assessed heterogeneity across SNPs; MR-Egger intercept analysis: tested for horizontal pleiotropy; leave-one-out analysis: evaluated the influence of individual SNPs on the overall results; Steiger test: verified that genetic variants primarily affected the exposure rather than the outcome. By integrating multiple statistical methods and comprehensive sensitivity analyses, this study provides a rigorous and consistent evaluation of the inferred causal relationships, ensuring the reliability of the conclusions.

## Results

### Overview of genetic instruments and Mendelian randomization method

Figure 1 illustrates our study flowchart, which aims to investigate the causal relationships between plasma metabolites and four liver disease subtypes: liver fibrosis/cirrhosis, hepatoma, hepatic failure, and metabolic liver disease. Table S2 provides detailed information on the utilization of 8,777 SNPs for plasma metabolite and metabolic ratios, with F-statistics varying between 20.69 and 99.88. Specifically, for hepatopathy, we analyzed 36 SNPs, comprising eight SNPs for fibrosis/cirrhosis, seven SNPs for hepatoma, 15 SNPs for hepatic failure, and six SNPs for metabolic liver disease, with F-statistics ranging from 9,565.59 to 10,929.36.

### The impact of plasma metabolites on liver disease risk

**Fig. 2:**
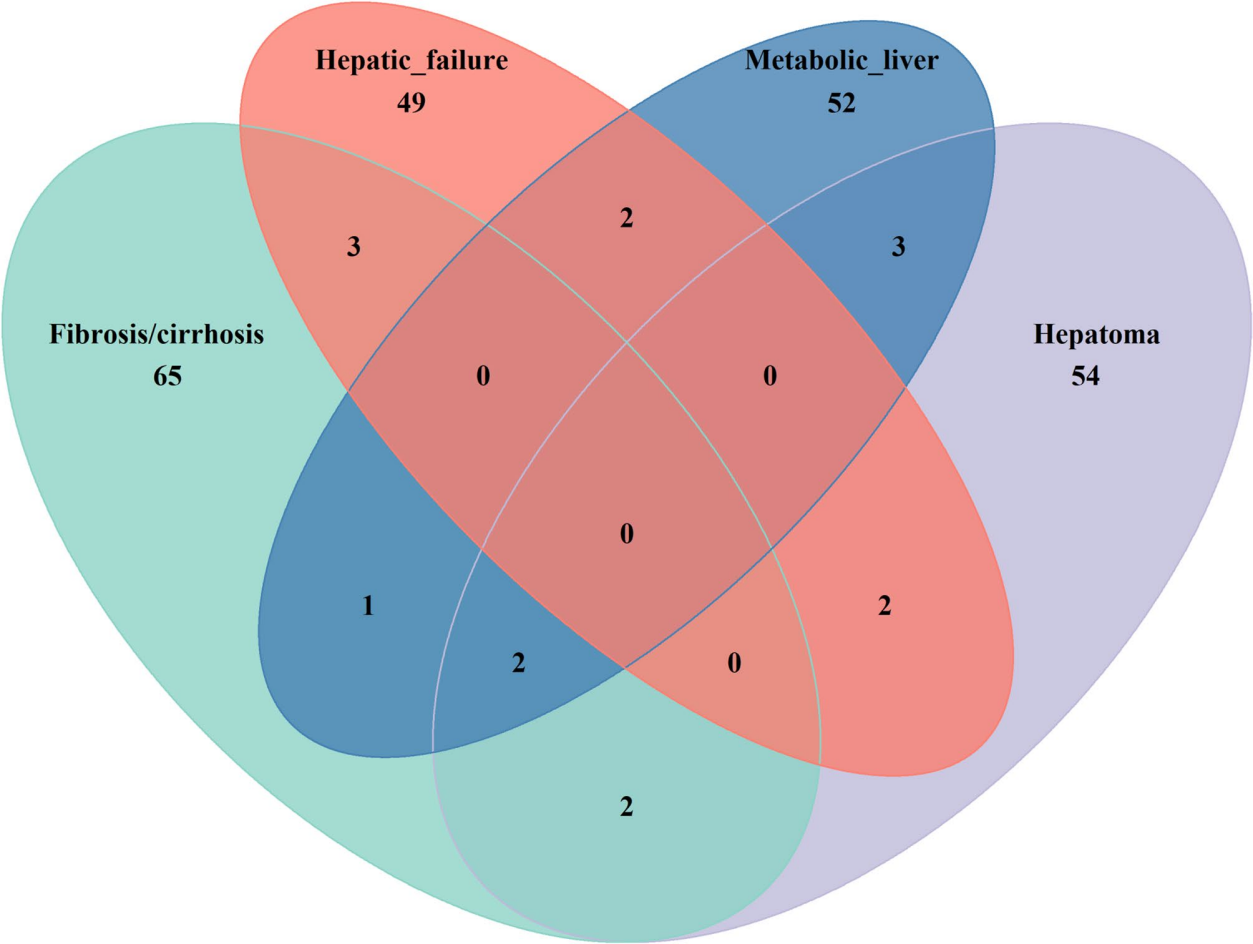
Multiple metabolic causes of liver disease intersect

**Fig. 3:**
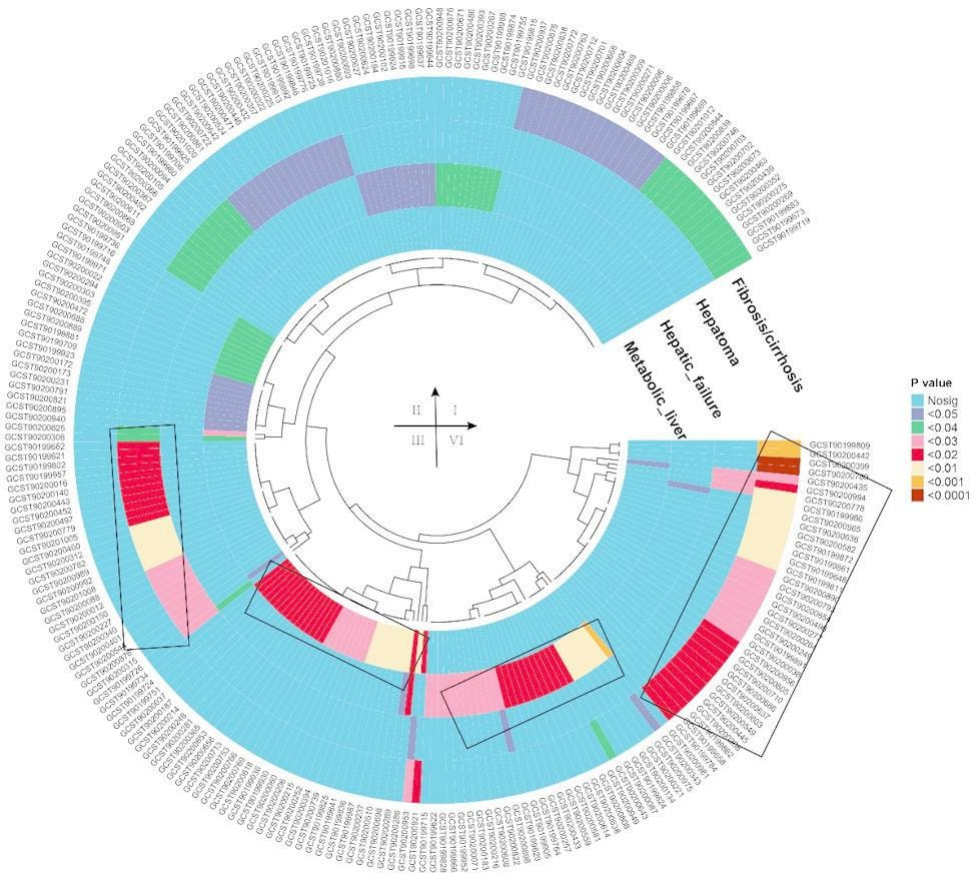
The results of a MR analysis. Color intensity reflects the strength of association (brown-red indicates a strong association, while blue signifies a weak or non-significant one). The outer circle lists metabolite IDs, and the inner circle represents various MR methods, with gradients indicating p-values. The metabolic indicators for fibrosis/cirrhosis are clustered in the upper-right and lower-right quadrants, while those for hepatoma are more widely distributed in the upper-left and lower-left quadrants. Hepatic failure indicators are concentrated in the upper and lower quadrants, and metabolic liver indicators are grouped in the middle-left and lower-left quadrants. This visualization underscores the unique and overlapping metabolic pathways that contribute to the etiology of liver diseases

The Venn diagram presented demonstrates a potential positive association (P_IVW_ < 0.05) between plasma metabolites and the risk of four liver diseases: fibrosis/cirrhosis, hepatic failure, metabolic liver disease, and hepatoma (Figs. 2 and 3 and Table S3). Among these diseases, 252 unique metabolic factors were identified. Specifically, 73 factors were associated with fibrosis/cirrhosis, 56 with hepatic failure, 60 with metabolic liver disease, and 63 with hepatomas. A detailed analysis revealed significant overlaps in metabolic factors among different liver diseases, including three common factors between fibrosis/cirrhosis and hepatic failure: eugenol sulfate levels, X-24,951 levels, and N-formylmethionine levels (Table S4). Additionally, two factors are shared between hepatic failure and metabolic liver disease: allantoin and N-acetylmethionine levels. Furthermore, five factors are present in both metabolic liver disease and hepatoma: the glucose-to-mannose ratio, gamma-glutamylhistidine levels, mannose levels, butyrate/isobutyrate (4:0) levels, and X-24,456 levels. Lastly, four factors overlap between fibrosis/cirrhosis and hepatoma, including the glucose-to-mannose ratio, glutamine-to-alanine ratio, mannose levels, and pyruvate to 3-methyl-2-oxobutyrate ratio.

**Fig. 4:**
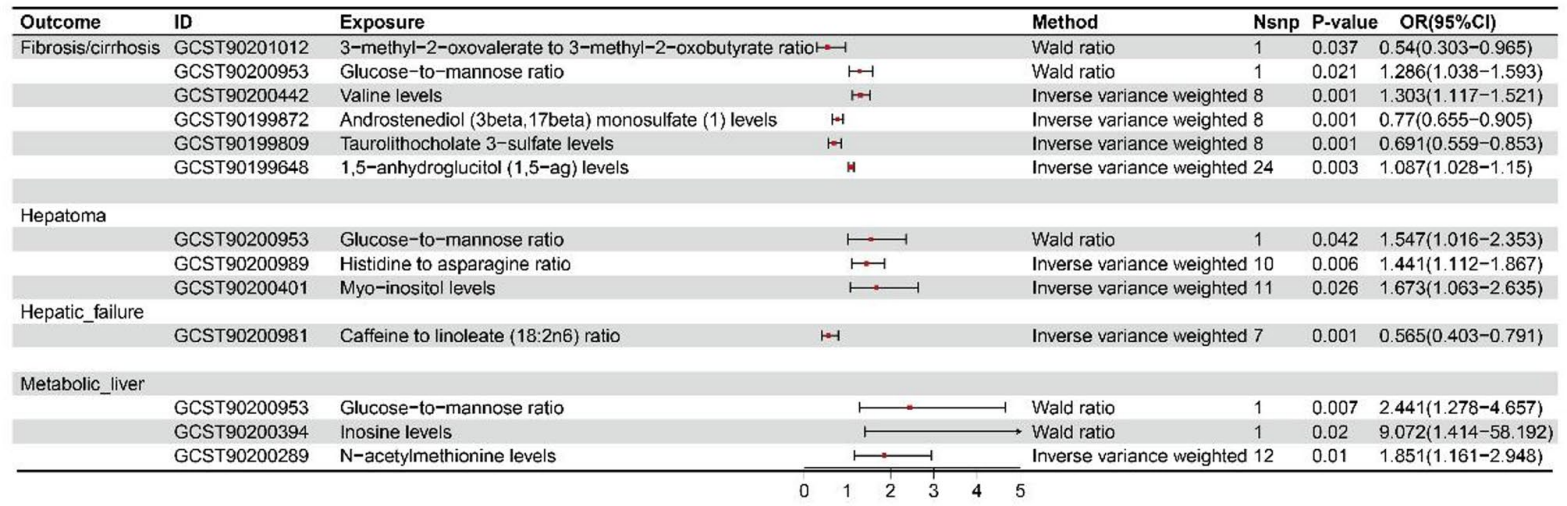
Reliable plasma biomarkers for liver disease

This study identified a range of plasma metabolites as potential biomarkers for various liver disease subtypes and demonstrated strong causal associations between these metabolites and liver disease risk (Fig. 4 and Table S5). Specifically, for fibrosis/cirrhosis, the study found positive associations with an increased glucose-to-mannose ratio (P_Wr_=0.021, OR_Wr_=1.286, 95% CI: 1.038–1.593), higher valine levels (P_IVW_=0.001, OR_IVW_=1.303, 95% CI: 1.117–1.521), and elevated 1,5-anhydroglucitol levels (P_IVW_=0.003, OR_IVW_=1.087, 95% CI: 1.028–1.15). Conversely, negative associations were observed between a higher ratio of 3-methyl-2-oxovalerate to 3-methyl-2-oxobutyrate (P_Wr_=0.037, OR_Wr_=0.54, 95% CI: 0.303–0.965), lower levels of androstenediol monosulfate (P_IVW_=0.001, OR_IVW_=0.77, 95% CI: 0.655–0.905), and reduced taurolithocholate 3-sulfate levels (P_IVW_=0.001, OR_IVW_=0.691, 95% CI: 0.559–0.853).

In hepatoma, positive associations were found with an increased glucose-to-mannose ratio (P_Wr_=0.042, OR_IVW_=1.547, 95% CI: 1.016–2.353), a higher histidine-to-asparagine ratio (P_IVW_=0.006, OR_IVW_=1.441, 95% CI: 1.112–1.867), and elevated myo-inositol levels (P_IVW_=0.026, OR_IVW_=1.673, 95% CI: 1.063–2.635). For hepatic failure, a negative association was observed with a lower caffeine-to-linoleate ratio (P_IVW_=0.001, OR_IVW_=0.565, 95% CI: 0.403–0.791).

Lastly, in metabolic liver disease, positive associations were found with an increased glucose-to-mannose ratio (P_Wr_=0.007, OR_Wr_=2.441, 95% CI: 1.278–4.657), higher inosine levels (P_Wr_=0.02, OR_Wr_=9.072, 95% CI: 1.414–58.192), and elevated N-acetylmethionine levels (P_IVW_=0.01, OR_IVW_=1.851, 95% CI: 1.161–2.948). These findings emphasize the critical role of plasma metabolites in metabolic mechanisms driving liver disease development and progression.

### Forward sensitivity analyses

The sensitivity analysis in Table S6 shows the strong causal link between plasma metabolites and various liver conditions. This shows that Qpval ≥ 0.05, suggesting no significant heterogeneity in the positive causal analysis, ensuring consistency among the instrumental variables. This finding strengthens the reliability of our findings. Moreover, Eggerpval ≥ 0.05 indicates no significant horizontal pleiotropy, supporting the robustness of the MR results. Leave-one-out analysis revealed that most SNPs had p-values below 0.05, statistically confirming their significant association with chronic liver diseases such as fibrosis, cancer, failure, and metabolic disorders (Table S7).

### The impact of chronic liver diseases on plasma metabolites and metabolic ratios

**Fig. 5:**
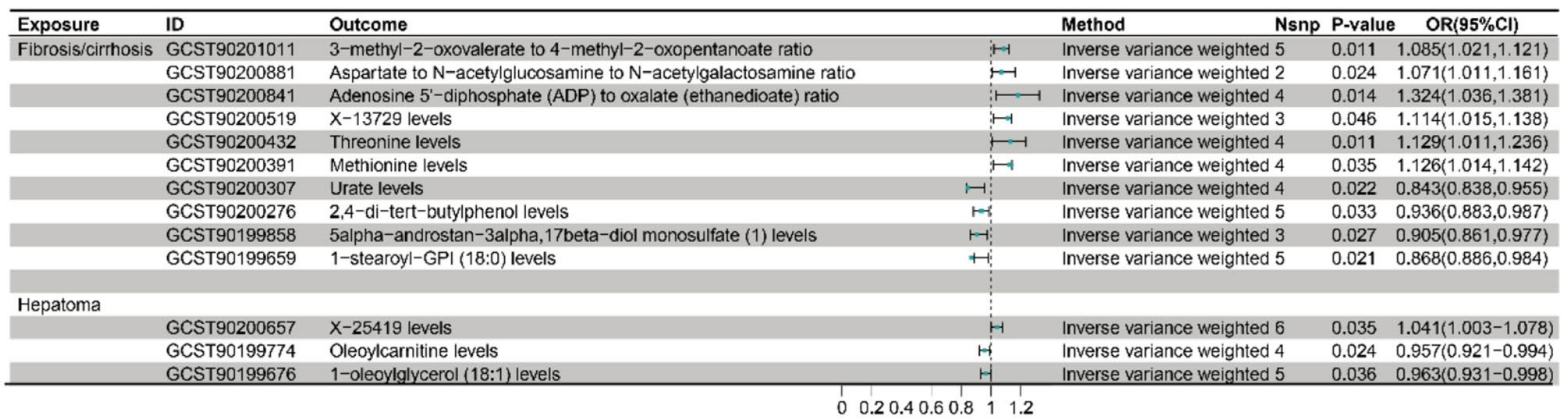
Changes in plasma metabolism due to liver disease

In this phase of the study, chronic liver disease subtypes were evaluated to determine potential associations with specific plasma metabolites and metabolic ratios (Fig. 5). The instrumental variables of hepatopathy and the reverse MR results for blood metabolites and hepatopathy are shown in Table S8 and Table S9. For fibrosis/cirrhosis, six metabolites showed positive associations: an increased ratio of 3-methyl-2-oxovalerate to 4-methyl-2-oxopentanoate (P_IVW_=0.011, OR_IVW_=1.085, 95% CI: 1.021–1.121), an elevated aspartate to N-acetylglucosamine to N-acetylgalactosamine ratio (P_IVW_=0.024, OR_IVW_=1.071, 95% CI: 1.011–1.161), a higher adenosine 5’-diphosphate (ADP) to oxalate ratio (PIVW = 0.014, ORIVW = 1.324, 95% CI: 1.036–1.381), increased X-13,729 levels (P_IVW_=0.046, OR_IVW_=1.114, 95% CI: 1.015–1.138), higher threonine levels (P_IVW_=0.011, OR_IVW_=1.129, 95% CI: 1.011–1.236), and elevated methionine levels (P_IVW_=0.035, OR_IVW_=1.126, 95% CI: 1.014–1.142).

Conversely, four metabolites showed negative associations: lower urate levels (P_IVW_=0.022, OR_IVW_=0.843, 95% CI: 0.838–0.955), decreased 2,4-di-tert-butylphenol levels (P_IVW_=0.033, OR_IVW_=0.936, 95% CI: 0.883–0.987), lower 5alpha-androstan-3alpha,17beta-diol monosulfate (1) levels (P_IVW_=0.027, OR_IVW_=0.905, 95% CI: 0.861–0.977), and reduced 1-stearoyl-GPI (18:0) levels (P_IVW_=0.021, OR_IVW_=0.868, 95% CI:0.886–0.984).

For hepatoma, one metabolite showed a positive association with increased X-25,419 levels (P_IVW_=0.035, OR_IVW_=1.041, 95% CI: 1.003–1.078), while two metabolites showed negative associations with lower oleoylcarnitine levels (P_IVW_=0.024, OR_IVW_=0.957, 95% CI: 0.921–0.994) and decreased 1-oleoylglycerol (18:1) levels (P_IVW_=0.036, OR_IVW_=0.963, 95% CI: 0.931–0.998). These findings provide insights into the metabolic mechanisms underlying the progression of chronic liver disease.

### Reverse sensitivity analyses

Our analysis indicated that fibrosis/cirrhosis was associated with six metabolites that exhibited positive correlations (such as Threonine and Methionine) and four metabolites that showed negative correlations (including urate and 1-stearoyl-GPI) (Table S8-10). The absence of significant heterogeneity, as evidenced by Q-pval values ≥ 0.05 or greater, confirms the consistency and reliability of these results. Furthermore, the lack of significant horizontal pleiotropy, with Egger-pval values also at 0.05 or greater, further validates the robustness of the findings related to fibrosis/cirrhosis. In hepatoma, one metabolite, X-25,419, demonstrated a positive correlation, whereas two metabolites, oleoylcarnitine and 1-oleoylglycerol, exhibited negative correlations. The reliability of these hepatoma-related results is supported by Q-pval values ≥ 0.05; however, Egger-Pval analysis was not applicable in this context. Additionally, the leave-one-out analysis revealed that most SNPs had p-values above 0.05, when considering the associations between liver fibrosis/cirrhosis and plasma metabolites or ratios, while some SNPs showed similar results for liver cancer (Table S11).

## Discussion

This study conducted a comprehensive investigation of the causal relationships between plasma metabolites, metabolic ratios, and chronic liver diseases using both forward and reverse MR analyses. Forward analysis identified 13 plasma metabolites and metabolic ratios significantly associated with liver fibrosis/cirrhosis, liver cancer, liver failure, and metabolic liver failure. Reverse analysis revealed that liver fibrosis/cirrhosis and liver cancer were causally linked to 10 and 3 plasma metabolite and metabolic ratios, respectively. The results were robust with no significant heterogeneity or pleiotropy detected, reinforcing the reliability of the findings.

Our study underscores the pivotal role of glucose metabolism disturbances, particularly glycolysis upregulation, in promoting liver fibrosis progression via hepatic stellate cell activation [20]. Additionally, androstenediol (3beta, 17beta) monosulfate may offer protective properties against fibrosis by enhancing antioxidative detoxification and modulating 11β-HSD1 [21, 22]. Furthermore, taurolithocholate 3-sulfate, a bile acid metabolite, negatively correlates with liver fibrosis by exerting anti-inflammatory and anti-fibrotic effects through FXR and TGR5 pathways [23, 24]. An elevated concentration of valine, a component of branched-chain amino acids (BCAAs), correlates with a heightened risk of fibrosis, whereas the ratio of 3-methyl-2-oxovalerate to 3-methyl-2-oxobutyrate demonstrates a protective effect [25]. The conflicting role of valine in fibrosis—anti-fibrotic in some experimental studies yet pro-fibrotic in our MR analysis—may be explained by several factors. First, although valine supplementation has been shown to suppress hepatic stellate cell activation in cellular and animal models [26], these findings may not directly extend to human populations. Second, our study focuses on systemic metabolite levels in blood, whereas prior work has emphasized intrahepatic valine dynamics. Blood concentrations may not fully correspond to tissue-level processes, which could contribute to divergent interpretations. Third, genetic pleiotropy may partly contribute to this discrepancy. Although we applied MR methods (MR-Egger and weighted median) to account for horizontal pleiotropy, and obtained consistent effect estimates, we cannot fully exclude the possibility that valine-associated genetic variants influence fibrosis through alternative biological pathways [27]. In addition, the potential opposing effects of valine and its metabolites could be complicated by the methodological heterogeneity across studies, including differences in population characteristics, amino acid sources, and analytical techniques [28, 29]. For instance, a higher ratio of 3-methyl-2-oxovalerate (from isoleucine) to 3-methyl-2-oxobutyrate (from valine) was associated with reduced fibrosis risk, suggesting that efficient valine catabolism may be protective while valine accumulation itself could be detrimental. Thus, the metabolic fate of valine—might influenced by genetic background, physiological context, and metabolic capacity—likely determines its net effect on fibrosis. In summary, the contradictory results emphasize the context-dependent role of valine and underscore the importance of considering metabolite-specific effects and analytical limitations when interpreting its association with fibrosis.

Our results revealed a positive correlation between elevated glucose-to-mannose ratio and the risk of fibrosis/cirrhosis. This increase in the glucose-to-mannose ratio may result from increased glucose and decreased mannose levels. High blood glucose levels have been shown to promote the activation of ADAM10 and proteolytic cleavage of gp130, leading to increased secretion of soluble gp130 (sgp130) from hepatic stellate cells (HSCs) along with IL-6, which is strongly associated with liver stiffness and histological evidence of liver fibrosis [30]. Additionally, high glucose levels may generate advanced glycation end products (AGEs) through non-enzymatic reactions [31]. AGEs enhance ECM production by inhibiting cell death in TGF-β1-activated HSCs and mediating aberrant crosslinking in the liver extracellular matrix, ultimately contributing to the development of fibrosis/cirrhosis [32, 33].

In metabolic liver disease, elevated levels of glucose-to-mannose ratio, inosine, and N-acetylmethionine are linked to increased disease risk. AGEs can contribute to insulin resistance (IR) by causing oxidative stress and chronic inflammation, which are essential for the development of metabolic dysfunction-associated steatotic liver disease (MASLD) [31, 34]. During IR, insulin’s ability to suppress gluconeogenesis is weakened, while hepatic lipogenesis increases uncontrollably [34, 35]. The absence of key molecules in the hepatic insulin signaling pathway (such as INSR/IRS/AKT) can inhibit the ability of insulin to promote liver fatty acid synthesis [34]. Despite this, increased gluconeogenesis provides substrates for liver lipid synthesis, leading to increased hepatic lipid deposition [34]. In addition, mannose, although not a significant energy source in humans, is necessary for protein glycosylation and can benefit high-fat diet (HFD) mice by reducing weight gain, fat mass, liver steatosis, and improving glucose tolerance [36]. Therefore, reduced mannose levels may contribute to MASLD progression.

In our study, the glucose-to-mannose ratio, histidine-to-asparagine ratio, and myo-inositol levels were found to increase significantly in individuals with hepatoma, consistent with previous research [37, 38]. This increase is linked to the altered metabolism of glucose and amino acids, which are crucial for the growth and survival of hepatoma cells [37, 38]. Specifically, glucose in hepatoma cells leads to degradation of the tumor suppressor p53, enhancing the Warburg effect and allowing tumors to grow [39]. AGEs also change the structure of collagen and increase ECM elasticity, aiding HCC cell growth and spreading through the integrin-β1–tensin-1–YAP pathway [40]. Mannose, like glucose, enters cells but accumulates inside mannose-6-phosphate, which then disrupts the metabolism of glucose in processes such as glycolysis, pentose phosphate pathway, and glycan synthesis [41]. Mannose exhibits multifaceted antitumor effects through both direct tumor cell inhibition and immunomodulatory mechanisms. When combined with conventional chemotherapy, it reduces anti-apoptotic Bcl-2 family protein levels, sensitizing tumor cells to death and suppressing tumor growth, and its administration could constitute a simple, safe, and selective therapy for multiple cancer types, as shown by Gonzalez et al. [41]. Furthermore, D-mannose enhances antitumor immunity by promoting stem-like properties in T cells through metabolic reprogramming and OGT-mediated O-GlcNAcylation of β-catenin, which sustains Tcf7 expression and epigenetic stemness [42]. Additionally, in ovarian cancer models, oral mannose supplementation enriches Faecalibaculum rodentium, which suppresses tumor progression and enhances antitumor immunity by expansion of progenitor-exhausted CD8 + T cells [43]. These findings highlight mannose’s broad therapeutic potential, demonstrating its ability to improve chemotherapy response and amplify immunotherapy outcomes across multiple cancer types through metabolic and immune modulation. In addition, previous researches have identified the glucose-to-mannose ratio as a risk factor for HER2-positive breast cancer, and its level in serum shows promise as a potential biomarker for ovarian cancer [44], implying its potential as an early diagnostic biomarker.

Oleoylcarnitine, a metabolite that accumulates following suppression of fatty acid β-oxidation, has been shown to promote hepatocarcinogenesis through STAT3 activation in the context of obesity-driven HCC [45]. Our results indicate that HCC may exert a causal effect on reducing oleoylcarnitine concentration, which may be explained by heterogeneity in tumor stage or subtype. HCC exhibits considerable diversity due to variations in underlying etiologies (e.g., obesity, viral infection, alcohol consumption), tumor microenvironments, and ethnic backgrounds [46]. Therefore, the role of oleoylcarnitine in HCC may vary depending on the specific etiological context. Furthermore, metabolic reprogramming evolves during HCC progression: early stages prioritize biosynthetic pathways and nutrient acquisition, while advanced stages may develop altered metabolic dependencies, such as enhanced oxidative phosphorylation or stress resistance mechanisms [47]. Thus, the context-specific nature of metabolic vulnerabilities may account for these divergent findings.

A higher caffeine-to-linoleate ratio was associated with a decreased risk of hepatic failure, whereas a lower ratio indicated an increased risk. Coffee consumption appears to reduce liver enzyme levels and exert a protective effect against complications in patients with advanced liver disease [48], implying its role in liver failure. This finding highlights the complex interplay among caffeine metabolism, fatty acid metabolism, and liver function. These results provide valuable insights for future research endeavors to explore metabolic mechanisms and their potential ramifications in liver diseases.

In reverse causal analysis, fibrosis/cirrhosis affected key metabolic pathways such as purine metabolism, aromatic metabolism, steroid metabolism, and glycerophospholipid metabolism, leading to reduced levels of metabolites such as urate, 2,4-di-tert-butylphenol, 5alpha-androstan-3alpha,17beta-diol monosulfate (1), and 1-stearoyl-GPI (18:0). These changes align with those of previous studies on metabolic disruptions [49, 50], although specific metabolite overlaps have been inconsistent across studies. HCC also causes significant disturbances in acylcarnitine levels, which are crucial for β-oxidation of fatty acids and mitochondrial function; however, there is conflicting evidence regarding acylcarnitine levels in HCC, with some studies reporting decreases [51] and others increases [52, 53]. These findings underscore the complexity of metabolic alterations in HCC and highlight the need for further research.

This study leverages bidirectional MR to explore causal links between plasma metabolites and chronic liver diseases, showing strengths such as the use of multiple MR methods, comprehensive metabolite coverage (1,400 metabolites), and sensitivity analyses to ensure result stability. However, this study had certain limitations. It is important to note that our results are derived from European/Finnish cohorts, which may limit their generalizability to other ethnic groups. The broader application of these findings necessitates validation in more diverse populations. Small SNP sample sizes in some analyses could weaken the statistical power. An exclusive focus on plasma metabolomics, excluding proteomics or transcriptomics, limits a holistic view of liver disease mechanisms. Despite MR reducing confounding, liver disease complexity and metabolite pleiotropy may still cause residual confounding factors. This study identified metabolite-disease links, but did not explore their biological mechanisms. Future research should prioritize population diversity, multi-omics data integration, and biological mechanism clarification, while enhancing MR techniques and validating biomarkers in clinical settings to minimize confounding and advance precision medicine in liver disease diagnostics and treatment.

## Conclusion

This study employed a bidirectional MR approach to investigate causal relationships between plasma metabolites and chronic liver conditions. Our findings underscore the significance of glucose metabolism in liver disease, revealing a correlation between an elevated glucose-to-mannose ratio and various liver conditions such as fibrosis/cirrhosis, metabolic liver disease, and hepatoma. These results contribute to a deeper understanding of the metabolic pathways involved and suggest promising directions for advancements in diagnostics and therapeutics.

## Supplementary Information

Supplementary Material 1

Supplementary Material 2

## Data Availability

No datasets were generated or analysed during the current study.

## Abbreviations

MR: Mendelian randomization
SNP: Single nucleotide polymorphism
IVW: Inverse variance weighted
HCC: Hepatocellular carcinoma
HSC: Hepatic stellate cells
ECM: Extracellular matrix
NAFLD: Non-alcoholic fatty liver disease
GWAS: Genome-wide association study
IR: Insulin resistance
MASLD: Metabolic dysfunction-associated steatotic liver disease
AGEs: Advanced glycation end products
