# Supplementary material for: Causal pathways of plasma metabolites: unveiling metabolic associations in liver diseases
Source: Nutr Metab (Lond). 2025 Nov 3;22:130. doi: 10.1186/s12986-025-01017-9 (PMC12581490; doi:10.1186/s12986-025-01017-9)
Supplement: Supplementary file 2 — Supplementary Material 2 [file 12986_2025_1017_MOESM2_ESM.docx]

**Supplementary Material**

Supplementary Information: TableS1-S11.

Table S1. List of the identification for each of the 1400 blood metabolites.

Table S2. The instrumental variable of the 1400 blood metabolites.

Table S3. The disease-specific metabolic clusters.

Table S4. Significant overlaps in metabolic factors among different liver diseases

Table S5. The MR results of blood metabolites and hepatopathy.

Table S6. Sensitivity analysis of blood metabolites and hepatopathy

Table S7. leave-one-out test of blood metabolites and hepatopathy.

Table S8. The instrumental variable of hepatopathy

Table S9. The reverse MR results of blood metabolites and hepatopathy.

Table S10. Sensitivity analysis of hepatopathy and blood metabolites.

Table S11. Leave-one-out test of hepatopathy and blood metabolites.
